# Narrowing Diurnal Temperature Amplitude Alters Carbon Tradeoff and Reduces Growth in C_4_ Crop Sorghum

**DOI:** 10.3389/fpls.2020.01262

**Published:** 2020-08-19

**Authors:** V. S. John Sunoj, P. V. Vara Prasad, Ignacio A. Ciampitti, Hanafey F. Maswada

**Affiliations:** ^1^Department of Agronomy, 2004 Throckmorton Plant Sciences Center, Kansas State University, Manhattan, KS, United States; ^2^State Key Laboratory of Conservation and Utilization of Subtropical Agro−bio−resources, College of Forestry, Guangxi University, Nanning, China; ^3^Department of Agricultural Botany, Faculty of Agriculture, Tanta University, Tanta, Egypt

**Keywords:** high night temperature, diurnal temperature amplitude, sorghum, night respiration, carbohydrates composition, growth

## Abstract

Effect of diurnal temperature amplitude on carbon tradeoff (photosynthesis vs. respiration) and growth are not well documented in C_4_ crops, especially under changing temperatures of light (daytime) and dark (nighttime) phases in 24 h of a day. Fluctuations in daytime and nighttime temperatures due to climate change narrows diurnal temperature amplitude which can alter circadian rhythms in plant, thus influence the ability of plants to cope with temperature changes and cause contradictory responses in carbon tradeoff, particularly in night respiration during dark phase, and growth. Sorghum [*Sorghum bicolor* (L.) Moench] is a key C_4_ cereal crop grown in high temperature challenging agro-climatic regions. Hence, it is important to understand its response to diurnal temperature amplitude. This is the first systematic investigation using controlled environmental facility to monitor the response of sorghum to different diurnal temperature amplitudes with same mean temperature. Two sorghum hybrids (DK 53 and DK 28E) were grown under optimum (27°C) and high (35°C) mean temperatures with three different diurnal temperature amplitudes (2, 10, and 18°C) accomplished by modulating daytime and nighttime temperatures [optimum daytime and nighttime temperatures (ODNT): 28/26, 32/22, and 36/18°C and high daytime and nighttime temperatures (HDNT): 36/34, 40/30, and 44/26°C]. After exposure to different temperature conditions, total soluble sugars, starch, total leaf area and biomass were reduced, while night respiration and specific leaf area were increased with narrowing of diurnal temperature amplitude (18 to 2°C) of HDNT followed by ODNT. However, there was no influence on photosynthesis across different ODNT and HDNT. Contradiction in response of foliar gas exchange and growth suggests higher contribution of night respiration for maintenance rather than growth with narrowing of diurnal temperature amplitude of ODNT and HDNT. Results imply that diurnal temperature amplitude has immense impact on the carbon tradeoff and growth, regardless of hybrid variation. Hence, diurnal temperature amplitude and night respiration should be considered while quantifying response and screening for high temperature tolerance in sorghum genotypes and comprehensive understanding of dark phase mechanisms which are coupled with stress response can further strengthen screening procedures.

## Introduction

Sorghum (*Sorghum bicolor* (L.) Moench) is an economically important C_4_ cereal crop for more than 500 million people around the globe due to its versatile usage as staple food, bioenergy, feed for livestock and industrial products (Maikasuwa and Ala, 2013; Tari et al., 2013; Ciampitti and Prasad, 2020). Sorghum occupies an important role in global food security along with other cereal crops such as wheat, millets, rice and maize (Ciampitti and Prasad, 2020; Maswada et al., 2020). Sorghum is relatively hardy crop as compared to C_3_ cereal crops, mainly grown in the semi-arid regions. In C_4_ crops, C_4_ photosynthetic pathway and related anatomical advantages enable them to tolerate warmer temperatures compared to C_3_ crops by maintaining photosynthesis *via*, concentrating CO_2_ and promoting the carboxylase activity of ribulose 1,5 bisphosphate carboxylate/oxygenase (Rubisco) and reducing the oxygenation activity (Sage and Kubien, 2007; Wang et al., 2012). However, anticipated more abrupt changes in weather, high daytime and nighttime temperatures can be important limiting factors for the growth, development and productivity of sorghum (Prasad et al., 2006b; Prasad et al., 2008a; Djanaguiraman et al., 2014).

Temperature and light cycles of light (day) and dark (night) phases of 24 h of a day synchronize circadian rhythms which includes subcellular level (gene expression, calcium signaling and activities of enzymes) and cell and tissue level (gas exchange, seed germination, hypocotyl elongation and behavior of leaves, flowers, and stomata) cycles to adjust with existing environmental conditions. Meanwhile, changes in temperature cycle, either increase or decrease in daytime or nighttime temperature, can adversely affect normal rhythmic response of plants (McClung, 2006; Srivastava et al., 2019). Negative impacts of high daytime and nighttime temperatures on different cereal crops has been well documented including in finger millet (Opole et al., 2018), maize (Sunoj et al., 2016; Lizaso et al., 2018), rice (Prasad et al., 2006a; Lin et al., 2020; Wang et al., 2020), and wheat (Prasad et al., 2008b; Prasad et al., 2015; Impa et al., 2018).

Each crop has cardinal or critical temperatures (minimum temperature, optimum temperature, and maximum temperature) with thresholds below (minimum temperature) or above (maximum temperature) in which no growth or development or physiological process occurs. Temperatures below and above optimum negatively impacts physiological process, traits, growth, development, and yield. Any growth temperature is the mean of daytime maximum and nighttime minimum temperatures. The same mean temperature can be obtained by different daily temperature (either daytime maximum and nighttime minimum temperatures or diurnal temperature range or amplitude). Identification of mean temperature threshold of growing season (different phenological stages) and responses of crop or process to different temperatures provides basis for quantifying impacts of climate change and adaptation options (Luo, 2011). For sorghum, the maximum critical threshold mean temperature is considered as 34°C (Hammer et al., 1993; Maiti, 1996), while, the optimum daytime and nighttime temperatures are 32 and 22°C, respectively (mean temperature of 27°C) (Prasad et al., 2006b). On a global scale prediction by inter-governmental panel for climate change (IPCC), pointed out a rise of 1.5 to 2°C in mean temperature by end of the 21^st^ century which is expected to be accompanied with more frequent heat waves and warmer nights (IPCC, 2018). These can negatively affect sorghum productivity, thereby a threat to global food security and economic status of farming community and allied industries.

In sorghum, high daytime and nighttime temperatures are reported to cause abortion of flowers, reduced pollen germination, phospholipid saturation of pollen, seed set, grain number and yield, biomass accumulation, photosynthesis, quantum yield of PS II, activities of antioxidant enzymes and increased reactive oxygen species (ROS), leaf night respiration, and thylakoid membrane damage (Prasad et al., 2006b; Prasad and Djanaguiraman, 2011; Djanaguiraman et al., 2014; Prasad et al., 2015; Singh et al., 2015; Sunoj et al., 2017; Djanaguiraman et al., 2018a; Narayanan et al., 2018). Even though, high daytime and nighttime temperatures negatively affect crops, nighttime temperature is more critical for crops grown on larger spatial or temporal scales. High nighttime temperature is negatively affecting at wide range of growth and developmental stages of crops and influencing responses of morphological, physiological, biochemical, and yield traits (Peng et al., 2004; Prasad et al., 2008a; Prasad et al., 2008b; Mohammed and Tarpley, 2009; Welch et al., 2010; Lobell et al., 2012; Djanaguiraman et al., 2013; Narayanan et al., 2015; Djanaguiraman et al., 2018b; Impa et al., 2018; Opole et al., 2018). Furthermore, due to climate change the rate of increase in daytime and nighttime temperatures can differ leading to lower diurnal amplitude (difference between daytime maximum and nighttime minimum temperatures) (Vose et al., 2005; Wang et al., 2017). Lower diurnal amplitude can negatively influence crop growth (Sunoj et al., 2016).

Studies to understand the effect of different diurnal temperature amplitudes with same mean temperature on C_4_ cereal crops are limited as compared to response of crops to either high daytime and nighttime temperatures (Myster and Moe, 1995; Sunoj et al., 2016). There are few studies to understand the effect of diurnal temperature amplitudes has been reported in different C_3_ crops such as cucumber (Xiong et al., 2011), orange (Bueno et al., 2012), eggplant, sweet pepper, tomato, melon, and watermelon (Inthichack et al., 2013; Inthichack et al., 2014; Matsuda et al., 2014). These studies revealed that diurnal temperature amplitude influence several morphological traits, foliar mineral composition, gas exchange and carbohydrate composition and metabolism. Hence, it is important to understand such responses in key food grain crops such as sorghum.

Important role of photosynthesis during light phase of a day in plant growth is beyond doubt. Response of light and dark phase gas exchange [day-time photosynthesis (CO_2_ assimilation) and night-time respiration (CO_2_ release), respectively] revealed that diurnal temperature amplitude induced night respiration during dark phase of a day has important role in carbon tradeoff (photosynthesis verses night respiration) which is one of the important factor determines the magnitude of growth along with other leaf traits and light interception (Hammer and Muchow, 1994; Ravi Kumar et al., 2009; Sunoj et al., 2016; Impa et al, 2018). Our study on C_4_ cereal crop maize [hybrid maize (DKC 47-27RIB, DEKALB, USA); Sunoj et al., 2016] systematically demonstrated the impact of different diurnal temperature amplitudes (2, 8, and 10°C) with optimum (30°C) and high (35°C) mean temperatures on vegetative growth. The study proved that, narrowing of diurnal temperature amplitude did not affected photosynthesis, but it increased leaf night respiration and reduced growth. At the same time, intensity of impact of narrowing of diurnal temperature amplitude on plant growth was varied among optimum and high mean temperature conditions.

Though, above study on maize provided a new insights on plant response to diurnal temperature amplitude and night respiration along with photosynthesis, it does not account the possibility of crop to crop variation and maintaining same or showing contrasting pattern of responses among different genotypes of same crop. It is also crucial to understand how sorghum, a relative more tolerant crop to abiotic stresses compared to maize, respond to these changes and current study is relevant as the sorghum grown in such regions which are experiencing and expecting higher temperatures in near future. Furthermore, based on available literatures, there are no reports dealing with response of sorghum to diurnal temperature amplitudes with an emphasis on carbon tradeoff and growth. Hence, this study was conducted to understand change in the pattern of response among different genotypes of same crop. To fill this knowledge gap, we performed current study with specific objective to quantify the impact of diurnal temperature amplitudes on growth, carbohydrate composition and carbon tradeoff in two sorghum hybrids. This research will help us to understand the dark phase processes, night respiration, and carbon utilization under temperature stress conditions. The results can be used to modify and strengthen the screening protocol for high temperature tolerance in sorghum. We hypothesize that dark phase (carbohydrate utilization *via* night respiration) and light phase (carbohydrate synthesis *via* photosynthesis) response are equally important and differentially influenced by diurnal temperature amplitude, thus affecting carbon trade-off and growth of sorghum genotypes.

## Materials and Methods

### Plant Material and Growth Conditions

The research was carried out using controlled environment facility at the Department of Agronomy, Kansas State University, Manhattan, Kansas, USA. Two *Sorghum bicolor* (L.) Moench hybrids (DK 53 and DK 28E; DEKALB, USA) were used for study. The sorghum seedlings were grown in 7 L plastic pots (21 cm height and 22 cm width) filled with growing medium (Metro mix 360 growing medium, Hummert International, Topeka, Kansas, USA). Pots were fertilized before sowing with macronutrients [35 g per pot; Osmocote classic; controlled release plant nutrients (14:14:14 NPK)], micronutrient (4 g per pot; Micro max; Hummert International, Topeka, Kansas, USA) and liquid iron (Iron 5%: Bonide products, Oriskany, New York, USA). Three seeds per pot were sown at a depth of 5 cm. To avoid incidence of sucking pest, 1 g of systemic insecticide Marathon {1% Imidacloprid, 1-[(6-Chloro-3-pyridinyl) methyl]-N-nitro-2-imidazolidin-mine; OHP Inc, Maryland, Pennsylvania, USA} was applied to each pot.

After sowing, pots were maintained inside growth chambers (Conviron Model PGR15; Winnipeg, MB, Canada) with controlled daytime and nighttime temperature conditions (32/22°C; optimum mean temperature 27°C), relative humidity (60% RH), 12 h of photoperiod (0600 to 1800 h) and photosynthetic active radiation (PAR) of 800 m mol m^-2^ s^-1^ at the plant canopy level using cool fluorescent lamps. To replicate the diurnal temperature variations in natural condition, a transition time of 7 h was set from day time maximum to night time minimum and *vice versa*. Air temperature was monitored at 10 min intervals throughout the experiment using HOBO data logger (Onset UTBi-001; TidbiT v2 Temperature logger; Bourne, Massachusetts, USA).

### Temperature Conditions

**Figure 1:**
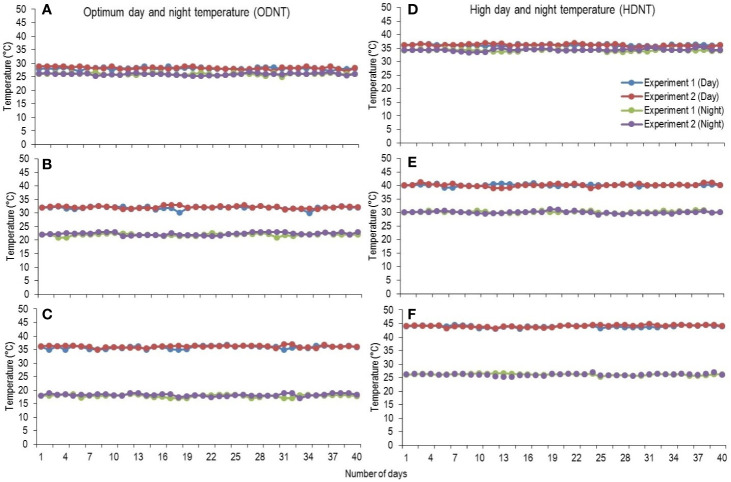
Maximum daytime (from 10:00 h to 14:00 h) and minimum nighttime temperature (from 22:00 h to 02:00 h) of two mean temperatures (27 and 35°C) with three diurnal temperature amplitudes (2 10, and 18°C) exposure lasting 40 days in controlled environment chambers. Mean optimum temperature [optimum mean daytime and nighttime temperature (ODNT)] 27°C: **(A)** Day/night temperature 28/26°C (diurnal temperature amplitude 2°C), **(B)** 32/22°C (diurnal temperature amplitude 10°C) and **(C)** 36/18°C (diurnal temperature amplitude 18°C). Mean high temperature [high mean day/night temperature (HDNT)] 35°C: **(D)** Day/night temperature 36/34°C (diurnal temperature amplitude 2°C), **(E)** 40/30°C (diurnal temperature amplitude 10°C) and **(F)** 44/26°C (diurnal temperature amplitude 18°C).

At third leaf stage [vegetative growth stage (GS-1); subdivision: third leaf collar stage (S1); Roozeboom and Prasad, 2016], sorghum seedlings were thinned to one plant per pot and growth chamber temperatures were adjusted to two different mean temperature conditions [optimum mean daytime and nighttime temperature (ODNT); 27°C and high mean daytime and nighttime temperature (HDNT); 35°C] with three different combinations of daytime and nighttime temperatures resulting in diurnal temperature amplitudes of 2, 10, and 18°C in different growth chambers (Sunoj et al., 2016). Three chambers were programmed with ODNT and other three chambers with HDNT. The diurnal temperature amplitudes (2, 10, and 18°C) with the ODNT was established by adjusting the daytime and nighttime temperatures (°C ± SD; standard deviation) to 28°C (± 0.3)/26°C (± 0.4), 32°C (± 0.5)/22°C (± 0.6), and 36°C (± 0.4)/18°C (± 0.6). Similarly, for achieving the different temperature amplitude with same HDNT, the dayime and nighttime temperatures were adjusted to 36°C (± 0.5)/34°C (± 0.3), 40°C (± 0.5)/30°C (± 0.5), and 44°C (± 0.4)/26°C (± 0.6) (**Figure 1**). Another independent experiment was repeated with similar growth conditions, genotypes and temperature conditions. A common set of growth, physiological and biochemical traits were recorded from both the experiments after exposing the seedlings to different temperature conditions for 40 days [reproductive growth stage (GS-2); subdivision: booting stage (S5); Roozeboom and Prasad, 2016].

### Foliar Gas Exchange and Photochemical Efficiency of PS II

Photosynthesis and night respiration were recorded from fully expanded mature leaves of sorghum seedlings using portable photosynthesis system (LI-6400 XT; LI-COR, Lincoln, NE, USA). On the final day (40^th^ day) of exposure to different temperature conditions, a minimum of six photosynthesis and night respiration measurements were recorded between 10:00 to 10:15 h and 22:00 to 22:15 h (growth chamber time settings), respectively. In order to maintain equal duration of exposure to day and night temperatures and light and dark phase, growth chamber programs were systematically adjusted to delay by 40 min between chamber to chamber from the actual time, which helped to over-come the time lag between chambers while measuring photosynthesis and leaf night respiration. This adjustment allowed us to capture the leaf photosynthesis and night respiration measurements exactly after 4 h exposure to light (PAR; 800 µmol m^-2^ s^-1^; 0600 h to 1000 h) and dark (PAR; 0 µmol m^-2^ s^-1^; 1800 to 2200 h) across all different temperature conditions. The CO_2_ concentration was set to 400 µmol mol^-1^ in the leaf chamber of the portable photosynthesis system and block temperature was adjusted to respective day (while measuring photosynthesis) and night (while measuring night respiration) temperature according to the growth chamber setting. The flow rate for photosynthesis measurement was 500 µmol s^-1^ and was adjusted to 100 µmol s^-1^ for measuring night respiration to minimize fluctuations (using the Li-6400/Li-6400XT; Portable Photosynthesis System; Version 6; LI-COR, Lincoln, Nebraska, USA; Sunoj et al., 2016). While measuring night respiration, sufficient care was taken to avoid exposure to PAR from any external sources and prior to recording night respiration, it was further confirmed by measuring the light with light sensor reader and six sensor quantum bar (field scout and light scout; Spectrum technologies, Inc., Aurora, Illinois, USA).

Maximum photochemical efficiency of PSII (*F*v/*F*m) of sorghum leaves was measured between 1000 and 1015 h after 30 min of dark adaptation using chlorophyll fluorometer (OS30p+; OptiSciences, Hudson, New Hampshire, USA) with light pulse intensity of 3000 µmol m^-2^ s^-1^ and pulse duration of 3 s (Sunoj et al., 2016).

### Carbohydrate Composition

At the end of temperature exposure, contents of total soluble sugars (TSS), reducing sugars (RS), non-reducing sugars (NRS), and starch were determined from the middle portion of fully opened mature leaves (without midrib). Tissue samples were collected from same leaves immediately after recording photosynthesis and immersed in liquid nitrogen and stored at -80°C until further analysis. Each sample was ground into powder using liquid nitrogen, homogenized thoroughly with ethanol (70%) and incubated at 70°C in a water bath for 30 min and filtered. The filtrate was used for the estimation of TSS [Phenol sulfuric acid method (Dubois et al., 1956)] and RS [Nelson Somogyi method (Somogyi, 1952)]. The NRS were estimated from the difference between TSS and RS (Malhotra and Sarkar, 1979). After filtration, the filter paper with the solid residue was kept in hot air oven at 50°C for drying and the dried residue was carefully collected and used for the estimation of starch by following anthrone method by Hedge and Hofreiter (1962).

### Total Above Ground Biomass Accumulation, Total Leaf Area, and Specific Leaf Area

Leaves were detached from the shoot and packed in zip lock bags to avoid drying and total leaf area (TLA) was measured using leaf area meter (LI 3100 area meter, LI-COR, Lincoln, Nebraska, USA). The total above ground biomass (TB) accumulation was calculated by cutting the plants from base and drying at 60°C in hot air oven until constant weight was obtained. The leaves that were detached to measure leaf area also included to calculate total biomass. Specific leaf area (SLA) was estimated by as ratio of total leaf area to the leaf dry weight (leaf area/leaf dry weight).

### Experimental Design and Statistical Analysis

Two independent controlled environmental chamber experiments were conducted with four biological replications per genotype per temperature conditions. The experimental design was randomized complete block (RCBD) for both experiments. Analysis of variance (ANOVA) was performed for all the measured traits using generalized linear model (GLM) in SPSS (SPSS Inc. Ver.16, USA) using temperatures, hybrids and their interactions as factors. The means were compared using Tukey’s honestly significant difference (HSD) range test. Correlations and regressions among different traits were performed using Sigma Plot (Systat Inc. Ver. 12.5, USA).

## Results

Across two indepentend experiments, there were significant (*P*<0.05) effect of temperature and hybrids when exposed to optimum (27°C; ODNT) and high (35°C; HDNT) daytime and nighttime temperatures with three diurnal temperature amplitudes (2, 10, and 18°C) on several physiological, biochemical and growth traits (**Tables 1** and **2**). Interaction effect of hybrids and temperature conditions was significant (*P*<0.05) on nine out of 10 measured traits (**Tables 1** and **2**). When exposed to different temperature conditions, both hybrids and measured traits were followed the same pattern in both experiments, which confirming the consistency and repeatability in results and response of hybrids. To demonstrate this, data from two independent experiments are independently presented here.

**Table 1:** Effect of two mean temperatures [27°C (optimum mean daytime and nighttime temperature; ODNT) and 35°C (high mean daytime and nighttime temperature; HDNT)] with three diurnal temperature amplitudes (2, 10, and 18°C) on growth and carbon tradeoff in sorghum hybrids (DK 53 and DK 28E).

Temperature conditions (T)		Total biomass(g plant^-1^)	Total leaf area(dm^2^ plant^-1^)	Specific leaf area(cm^2^ g^-1^DW)	Photosynthesis (µmol m^-2^ s^-1^)	Photochemical efficiency of PSII (*F*v/*F*m)	Night respiration (µmol m^-2^ s^-1^)
Experiment I							
Day/Night temperature	Temperature amplitude						
Optimum (ODNT; 27°C)							
28/26°C	2°C	116.6 ± 11.5^c^	89.21 ± 2.62^c^	187.6 ± 7.21^a^	24.31 ± 1.09^a^	0.795 ± 0.005^a^	0.499 ± 0.001^c^
32/22°C	10°C	228.6 ± 10.5^b^	110.3 ± 3.65^b^	176.4 ± 8.44^b^	23.22 ± 1.30^a^	0.790 ± 0.005^ab^	0.426 ± 0.002^e^
36/18°C	18°C	281.1 ± 11.0^a^	128.8 ± 5.21^a^	166.5 ± 6.35^c^	23.91 ± 1.55^a^	0.780 ± 0.005^cd^	0.347 ± 0.001^f^
Mean		208.8 ± 10.5	109.4 ± 3.6	176.8 ± 7.4	23.81 ± 1.5	0.788 ± 0.005	0.424 ± 0.001
High (HDNT; 35°C)							
36/34°C	2°C	79.57 ± 10.6^d^	75.83 ± 2.02^d^	217.9 ± 5.23^a^	22.79 ± 1.60^a^	0.770 ± 0.005^e^	0.802 ± 0.002^a^
40/30°C	10°C	110.5 ± 11.4^c^	80.68 ± 2.74^d^	195.2 ± 7.82^b^	22.77 ± 1.35^a^	0.775 ± 0.003^de^	0.575 ± 0.002^b^
44/26°C	18°C	127.8 ± 9.95^c^	105.7 ± 4.06^b^	183.7 ± 8.56^bc^	22.44 ± 1.32^a^	0.785 ± 0.003^bc^	0.476 ± 0.002^d^
Mean		106.0 ± 10.2**	87.41 ± 2.4**	198.9 ± 7.2*	22.67 ± 1.2^NS^	0.777 ± 0.003*	0.617 ± 0.002**
Hybrids (H)							
	DK 53	136.4 ± 11.1	90.35 ± 2.98	187.4 ± 7.80	22.78 ± 1.28	0.787 ± 0.004	0.542 ± 0.001
	DK 28E	178.4 ± 10.5**	106.5 ± 3.78**	188.3 ± 6.51^NS^	23.70 ± 1.46^NS^	0.778 ± 0.003 ^NS^	0.500 ± 0.002*
Probability values							
	T	<0.01	<0.01	<0.01	0.183	<0.05	<0.01
	H	<0.01	<0.01	0.738	0.059	<0.652	<0.01
	T x H	<0.01	<0.01	<0.05	0.089	<0.05	<0.01
Experiment II							
Optimum (ODNT; 27°C)							
28/26°C	2°C	109.8 ± 11.0^de^	82.05 ± 2.16^e^	197.5 ± 10.2^a^	23.60 ± 1.35^a^	0.795 ± 0.004^a^	0.521 ± 0.002^c^
32/22°C	10°C	206.5 ± 10.95^b^	118.2 ± 4.32^b^	179.2 ± 9.41^b^	22.75 ± 1.55^a^	0.780 ± 0.003^b^	0.425 ± 0.002^d^
36/18°C	18°C	249.7 ± 11.55^a^	132.0 ± 4.94^a^	165.3 ± 6.33^bc^	23.30 ± 1.51^a^	0.795 ± 0.003^a^	0.305 ± 0.003^f^
Mean		188.7 ± 10.2	110.7 ± 3.2	180.7 ± 8.1	23.22 ± 1.23	0.790 ± 0.003	0.417 ± 0.002
High (HDNT; 35°C)							
36/34°C	2°C	88.31 ± 8.55^e^	73.36 ± 2.12^f^	213.3 ± 11.4^a^	23.00 ± 1.90^a^	0.780 ± 0.003^b^	0.895 ± 0.002^a^
40/30°C	10°C	122.1 ± 9.80^cd^	90.66 ± 2.84^d^	199.6 ± 10.5^b^	23.80 ± 1.55^a^	0.774 ± 0.003^b^	0.535 ± 0.001^b^
44/26°C	18°C	138.4 ± 12.2^c^	105.4 ± 3.82^c^	182.9 ± 6.93^c^	22.91 ± 1.60^a^	0.775 ± 0.007^b^	0.420 ± 0.002^e^
Mean		116.2 ± 11.2**	89.79 ± 3.2**	198.6 ± 7.4*	23.24 ± 1.50^NS^	0.776 ± 0.005*	0.617 ± 0.002**
Hybrids (H)							
	DK 53	130.1 ± 11.2	91.56 ± 2.94	186.9 ± 6.9	23.15 ± 1.58	0.783 ± 0.003	0.537 ± 0.002
	DK 28E	174.8 ± 10.2**	109.0 ± 3.79**	192.3 ± 8.2 ^NS^	23.30 ± 1.57^NS^	0.783 ± 0.004^NS^	0.497 ± 0.002*
Probability values							
	T	<0.01	<0.01	<0.01	0.840	<0.05	<0.01
	H	<0.01	<0.01	<0.068	0.777	0.796	<0.05
	T x H	<0.01	<0.01	<0.05	0.576	<0.05	<0.01

Values in each column are mean of both sorghum hybrids (DK 53and DK 28E) with ± standard error under different day and night temperatures of ODNT and HDNT. Values followed by different letters indicates significant difference according to Tukey’s HSD (P<0.01; the test was conducted independently for each experiments). The mean values of ODNT and HDNT (irrespective of hybrids and three diurnal temperature amplitudes) and two sorghum hybrids (DK 53and DK 28E; irrespective of three diurnal temperature amplitudes of ODNT and CDNT) were compared using student’s t-test and * and ** corresponding to significance at P < 0.05 and P < 0.01, respectively. NS indicate non-significant.

**Table 2:** Effect of two mean temperatures [27°C (optimum mean daytime and nighttime temperature; ODNT) and 35°C (high mean daytime and nighttime temperature; HDNT)] with three diurnal temperature amplitudes (2, 10, and 18°C) on carbohydrate composition in sorghum hybrids (DK 53 and DK 28E).

Temperature conditions (T)		Total soluble sugars(mg g^-1^ DW)	Reducing sugars(mg g^-1^ DW)	Non-reducing sugars(mg g^-1^ DW)	Starch(mg g^-1^ DW)
Experiment I					
Day/Night temperature	Temperature amplitude				
Optimum (ODNT; 27°C)					
28/26°C	2°C	5.05 ± 0.06^c^	2.30 ± 0.08^b^	2.75 ± 0.09^e^	144.5 ± 7.37^bc^
32/22°C	10°C	6.25 ± 0.05^b^	2.15 ± 0.06^bc^	4.10 ± 0.05^c^	169.5 ± 6.55^a^
36/18°C	18°C	14.1 ± 0.05^a^	4.55 ± 0.07^a^	9.55 ± 0.07^a^	181.0 ± 5.10^a^
Mean		8.47 ± 0.05	3.00 ± 0.07	5.47 ± 0.08	165.0 ± 6.21
High (HDNT; 35°C)					
36/34°C	2°C	3.70 ± 0.08^d^	2.00 ± 0.07^c^	1.70 ± 0.06^f^	116.0 ± 5.20^d^
40/30°C	10°C	4.70 ± 0.07^c^	1.60 ± 0.07^d^	3.10 ± 0.05^d^	135.5 ± 5.15^c^
44/26°C	18°C	6.00 ± 0.08^b^	1.35 ± 0.06^e^	4.65 ± 0.07^b^	155.0 ± 6.40^b^
Mean		4.80 ± 0.08**	1.65 ± 0.06*	3.15 ± 0.06*	135.5 ± 5.5*
Hybrids (H)					
	DK 53	6.13 ± 0.13	2.13 ± 0.08	4.00 ± 0.07	137.3 ± 6.01
	DK 28E	7.13 ± 0.16*	2.52 ± 0.06*	4.62 ± 0.06*	163.2 ± 5.92**
Probability values					
	T	<0.01	<0.01	<0.01	<0.01
	H	<0.05	<0.05	<0.05	<0.01
	T x H	<0.01	<0.01	<0.01	<0.05
Experiment II					
Optimum DNT (27°C)					
28/26°C	2°C	5.00 ± 0.04^c^	3.25 ± 0.04^c^	1.75 ± 0.06^d^	141.0 ± 4.10^c^
32/22°C	10°C	6.60 ± 0.05^b^	3.75 ± 0.07^b^	2.85 ± 0.08^c^	169.5 ± 6.55^b^
36/18°C	18°C	11.95 ± 0.30^a^	5.05 ± 0.07^a^	6.90 ± 0.07^a^	193.5 ± 8.10^a^
Mean		7.85 ± 0.04	4.02 ± 005	3.83 ± 0.06	168.0 ± 6.5
High (HDNT; 35°C)					
36/34°C	2°C	3.85 ± 0.07^d^	2.20 ± 0.04^d^	1.65 ± 0.07^d^	112.0 ± 4.70^d^
40/30°C	10°C	4.75 ± 0.07^c^	1.75 ± 0.04^e^	3.00 ± 0.05^c^	137.5 ± 6.20^c^
44/26°C	18°C	6.65 ± 0.07^b^	1.50 ± 0.04^f^	5.15 ± 0.08^b^	161.0 ± 4.45^b^
Mean		5.08 ± 0.07**	1.82 ± 0.047**	3.27 ± 0.06^NS^	136.8 ± 0.05**
Hybrids (H)					
	DK 53	6.13 ± 0.08	2.87 ± 0.05	3.27 ± 0.06	146.0 ± 5.88
	DK 28E	6.80 ± 0.12*	2.97 ± 0.05*	3.83 ± 0.07*	158.8 ± 5.48*
Probability values					
	T	<0.01	<.01	<0.01	<0.01
	H	<0.05	<0.05	<0.05	<0.05
	T x H	<0.01	<0.01	<0.01	<0.01

Values in each column are mean of both sorghum hybrids (DK 53 and DK 28E) with ± standard error under different day and night temperatures of ODNT and HDNT. Values followed by different letters indicates significant difference according to Tukey’s HSD (P<0.01; the test was conducted independently for each experiments). The mean values of ODNT and HDNT (irrespective of hybrids and three diurnal temperature amplitudes) and two sorghum hybrids (DK 53and DK 28E; irrespective of three diurnal temperature amplitudes of ODNT and CDNT) were compared using student’s t-test and * and ** corresponding to significance at P < 0.05 and P < 0.01, respectively. NS indicate non-significant.

### Effects on Growth

**Figure 2:**
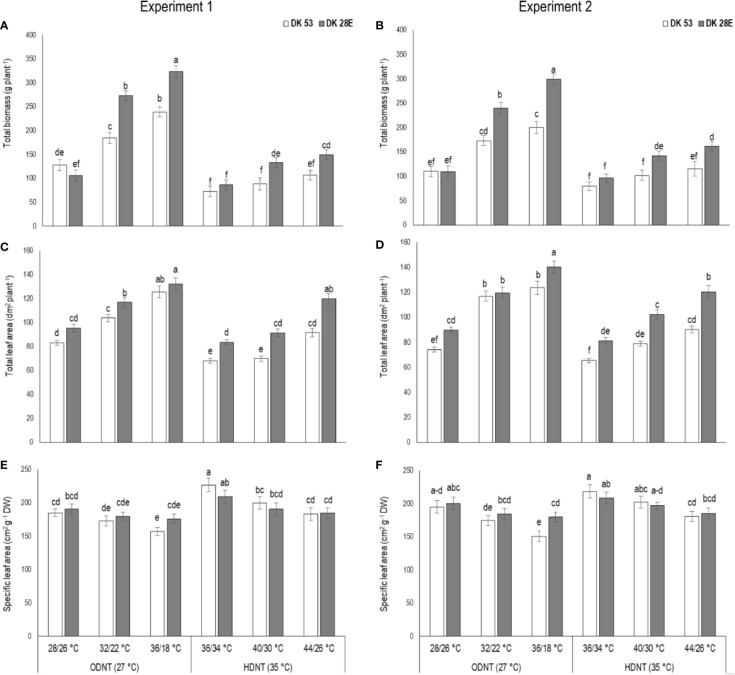
Effect of two mean temperatures (27 and 35°C) [27°C (optimum mean daytime and nighttime temperature; ODNT) and 35°C(high mean daytime and nighttime temperature; HDNT)] with three diurnal temperature amplitudes (2, 10, and 18°C) on **(A, B)** total biomass (TB), **(C, D)** total leaf area (TLA) and **(E, F)** specific leaf area (SLA) of two sorghum hybrids [DK 53 (white bar) and DK 28E (gray bar)]. Different letters in bar indicates significant difference according to Tukey’s HSD (*P*<0.01; the test was conducted independently for each experiments). Each bar is mean value with standard error. Dry weight (DW).

In spite of differences among hybrids and diurnal temperature amplitudes, growth of seedlings indicated by total aboveground biomass accumulation (TB) and total leaf area (TLA) were significantly (*P<*0.01) higher and specific leaf area (SLA) was significantly (*P<*0.05) lower under ODNT compared to HDNT (**Table 1**). At the same time, TB and TLA of both hybrids were reduced and SLA was increased with narrowing diurnal temperature amplitude (18 to 2°C) regardless of two mean temperatures (ODNT and HDNT) (**Table 1** and **Figure 2**). Seedlings exposed to ODNT with highest diurnal temperature amplitude (18°C) recorded higher TB and TLA; and lowest SLA, while lowest TB and TLA and higher SLA was observed under HDNT with narrow diurnal temperature amplitude (2°C) across different day and night conditions (**Table 1** and **Figure 2**).

Among the hybrids, DK 28E attained higher TB, TLA, and SLA across all the diurnal temperature amplitudes irrespective of both different mean temperatures, expect an opposite trend in TB and TLA at narrow diurnal temperature amplitude of 2°C (**Figure 2**). On an average of two experiments, as compared to narrow diurnal temperature amplitude (2°C) with ODNT and HDNT, highest percentage increase of 189% in TB was in DK 28E and 58% in TLA was in DK 53 under highest diurnal temperature amplitude (18°C) with ODNT, while highest percentage reduction of 19% in SLA was observed in DK 53 (**Table 3**).

**Table 3:** Percentage change in growth and carbon tradeoff in two sorghum hybrids (DK 53 and DK 28E) under diurnal temperature amplitutes of 10°C and 18°C compared to narrow diurnal temperature ampltude of 2°C with two mean temperatures [27°C (optimum mean daytime and nighttime temperature; ODNT) and 35°C (high mean daytime and nighttime temperature; HDNT)].

Temperature conditions/Hybrids		Total biomass	Total leaf area	Specific leaf area	Photosynthesis	Photochemical efficiency of PSII (*F*v/*F*m)	Night respiration
DK 53							
Day/Night temperature	Temperature amplitude	Percentage (%) change					
Optimum (ODNT; 27°C)							
32/22°C	10°C	50	40	-9	-4.3	-1.9	-15
36/18°C	18°C	85	58	-19	-0.5	-1.3	-37
High (HDNT; 35°C)							
40/30°C	10°C	25	12	-7	7.8	0.5	-30
44/26°C	18°C	45	36	-9	4.3	0.6	-48
DK 28E							
Optimum (ODNT; 27°C)		Percentage (%) change					
32/22°C	10°C	138	28	-10	-3.8	-0.6	-18
36/18°C	18°C	189	47	-18	-2.4	-0.6	-36
High (HDNT; 35°C)							
40/30°C	10°C	50	18	-7	-3.8	-0.6	-39
44/26°C	18°C	70	46	-11	-5.7	0.6	-47

Values in each column are mean of percentage change of two experiments.

### Effects on Carbon Tradeoff and Photochemical Efficiency of PSII

**Figure 3:**
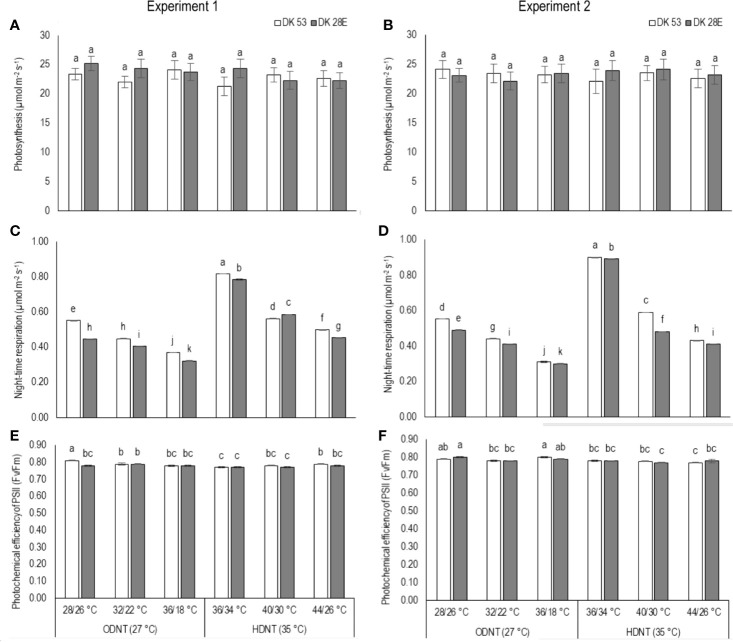
Effect of two mean temperatures (27 and 35°C) [27°C (optimum mean daytime and nighttime temperature; ODNT) and 35°C (high mean daytime and nighttime temperature; HDNT)] with three diurnal temperature amplitudes (2, 10, and 18°C) on **(A, B)** photosynthesis (*P*_N_), **(C, D)** photochemical efficiency of PSII (*F*v/*F*m) and **(E, F)** night respiration of two sorghum hybrids [DK 53 (white bar) and DK 28E (gray bar)]. Different letters in bar indicates significant difference according to Tukey’s HSD (*P*<0.01; the test was conducted independently for each experiments). Each bar is mean value with standard error. Dry weight (DW).

There was a contradiction observed in carbon tradeoff (photosynthesis *vs.* night respiration) in both hybrids exposed to different temperature conditions. Photosynthesis was not significantly varied across different diurnal temperature amplitudes with different mean temperatures and among hybrids, while there was a distinct variation found in night respiration (**Tables 1** and **3** and **Figure 3**). The night respiration was significantly (*P<*0.01) higher across different diurnal temperature amplitudes with HDNT compared to diurnal temperature amplitudes with ODNT (**Table 1**). Night respiration was 37% and 48% less in DK 53 and 36% and 47% in DK 28E under 18°C diurnal temperature amplitudes with ODNT and HDNT, respectively, as compared to 2°C (**Table 3**). Regardless of different mean temperatures, magnitude of night respiration was increased with narrowing diurnal temperature amplitude, and among hybrids, DK 53 showed slightly higher increase than DK 28 (**Figure 3**). Meanwhile, photochemical efficiency of PSII (*F*v/*F*m) of both hybrids under different diurnal temperature amplitudes of HDNT and ODNT was not uniform in both experiments (**Tables 1** and **3**). HDNT slightly reduced *F*v/*F*m compared with ODNT (**Table 1**). Though there was a significant difference (*P<*0.05) among temperature conditions and its interaction with hybrids, values of *F*v/*F*m ranged between 0.77 to 0.81 across hybrids, temperature conditions, and experiments (**Figure 3**).

### Effects on Carbohydrate Composition of Sorghum Hybrids

**Figure 4:**
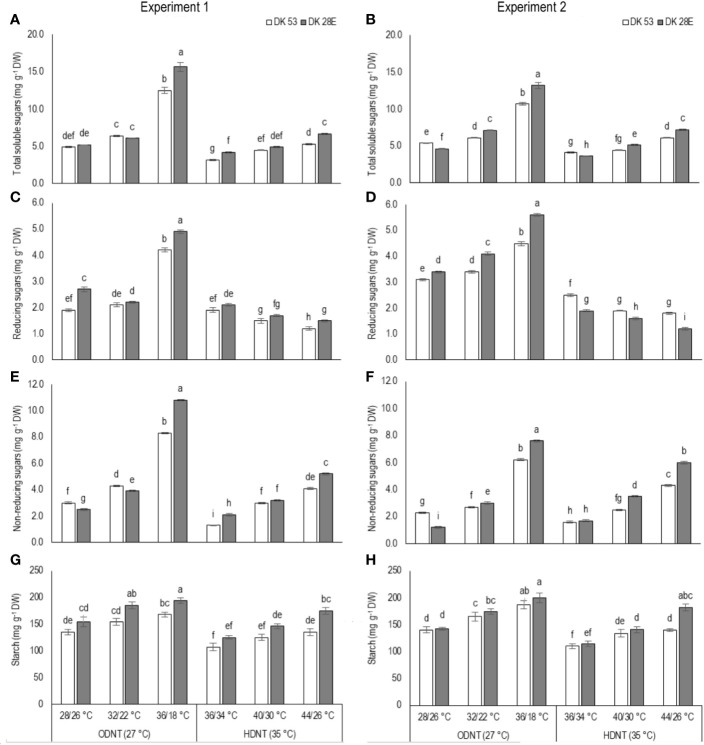
Effect of two mean temperatures (27 and 35°C) [27°C (optimum mean daytime and nighttime temperature; ODNT) and 35°C (high mean daytime and nighttime temperature; HDNT)] with three diurnal temperature amplitudes (2, 10, and 18°C) on **(A, B)** total soluble sugars (TSS), **(C, D)** reducing sugars (RS), **(E, F)** non-reducing sugars (NRS) and **(G, H)** starch of two sorghum hybrids [DK 53 (white bar) and DK 28E (gray bar)]. Different letters in bar indicates significant difference according to Tukey’s HSD (*P*<0.01; the test was conducted independently for each experiments). Each bar is mean value with standard error. Dry weight (DW).

In both experiments, content of total soluble sugars (TSS), reducing sugars (RS), non-reducing sugars (NRS) and starch of both hybrids were significantly (*P<*0.01) altered after the exposure to different temperature conditions (**Table 2**). TSS, NRS, RS, and starch were reduced with narrowing diurnal temperature amplitudes (18 to 2°C), except RS under HDNT which showed an opposite trend (**Figure 4**). Regardless of the diurnal temperature amplitudes, across both experiments, carbohydrate composition was decreased under HDNT compared with ODNT (**Table 2**).

On an average, among hybrids, percentage increase in majority of carbohydrate components were high in DK 28E than DK 53 under diurnal temperature amplitude of 18°C with ODNT (195% in TSS, 397% in NRS, and 33% in starch) and HDNT (78% in TSS, 195% in NRS, and 49% in starch) as compare to 2°C diurnal amplitude (**Table 4**). At the same time, an opposite trend in hybrid response was observed in RS (**Figure 4**).

**Table 4:** Percentage change in carbohydrate composition in two sorghum hybrids (DK 53 and DK 28E) under diurnal temperature amplitutes of 10 and 18°C compared to narrow diurnal temperature ampltude of 2°C with two mean temperatures [27°C (optimum mean daytime and nighttime temperature; ODNT) and 35°C (high mean daytime and nighttime temperature; HDNT)].

Temperature conditions/Hybrids		Total soluble sugars	Reducing sugars	Non-reducing sugars	Starch
DK 53					
Day/Night temperature	Temperature amplitude	Percentage (%) change			
Optimum (ODNT; 27°C)					
32/22°C	10°C	21	10	32	16
36/18°C	18°C	125	74	174	29
High (HDNT; 35°C)					
40/30°C	10°C	22	-23	90	19
44/26°C	18°C	56	-32	190	27
DK 28E					
Optimum (ODNT; 27°C)		Percentage (%) change			
32/22°C	10°C	35	3	86	21
36/18°C	18°C	195	72	397	33
High (HDNT; 35°C)					
40/30°C	10°C	28	-18	76	20
44/26°C	18°C	78	-33	195	49

Values in each column are mean of percentage change of two experiments.

### Relationship of Night Respiration With Growth Traits and Carbohydrates

**Figure 5:**
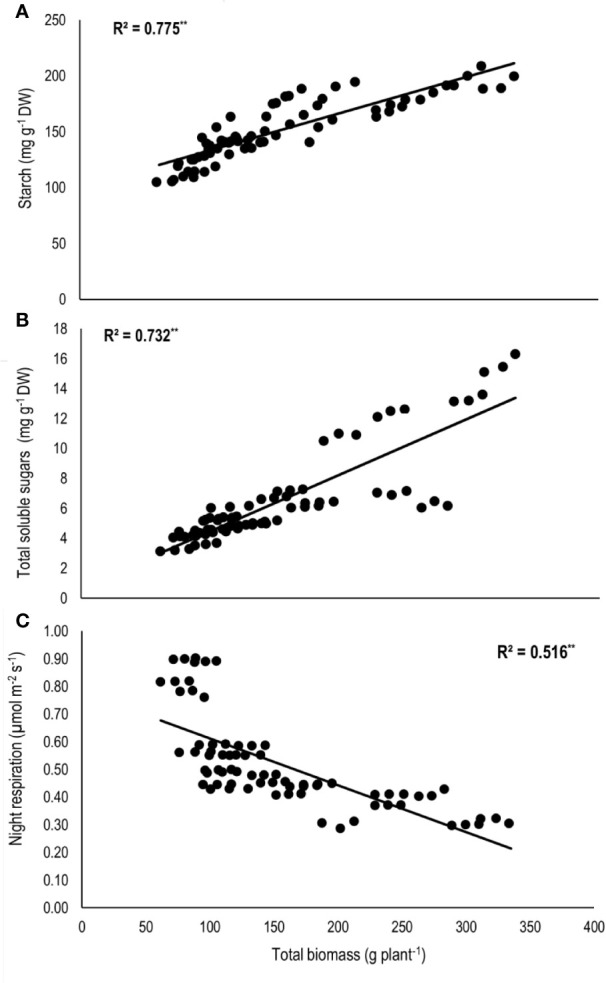
Relationship of total biomass (TB) with **(A)** starch, **(B)** total soluble sugars (TSS), and **(C)** night respiration of two sorghum hybrids (DK 53 and DK 28E) exposed to two mean temperatures (27 and 35°C) [27°C (optimum mean daytime and nighttime temperature; ODNT) and 35°C (high mean daytime and nighttime temperature; HDNT)] with three diurnal temperature amplitudes (2, 10, and 18°C). Coefficient of determination (R^2^) followed by ** significant at *P* < 0.01. Dry weight (DW).

**Figure 6:**
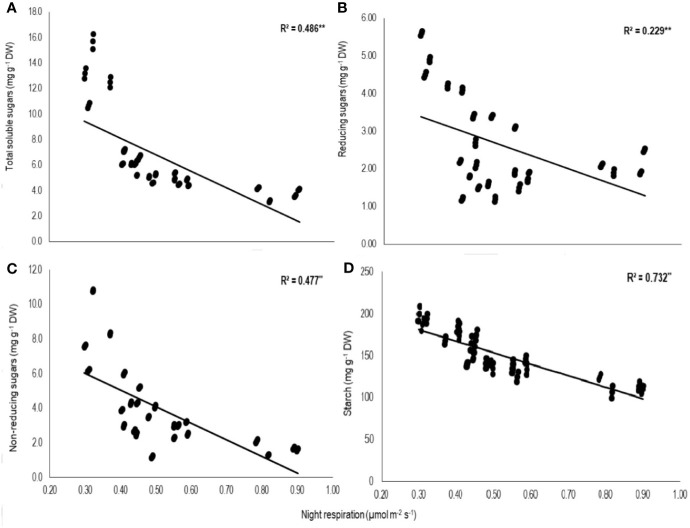
Relationship of night respiration with **(A)** total soluble sugars (TSS), **(B)** reducing sugars (RSS), **(C)** non-reducing sugars (NRS), and **(D)** starch of two sorghum hybrids (DK 53 and DK 28E) exposed to two mean temperatures (27 and 35°C) [27°C (optimum mean daytime and nighttime temperature; ODNT) and 35°C (high mean daytime and nighttime temperature; HDNT)] with three diurnal temperature amplitudes (2, 10, and 18°C). Coefficient of determination (R^2^) followed by ** significant at *P* < 0.01. respectively. Dry weight (DW).

**Figure 7:**
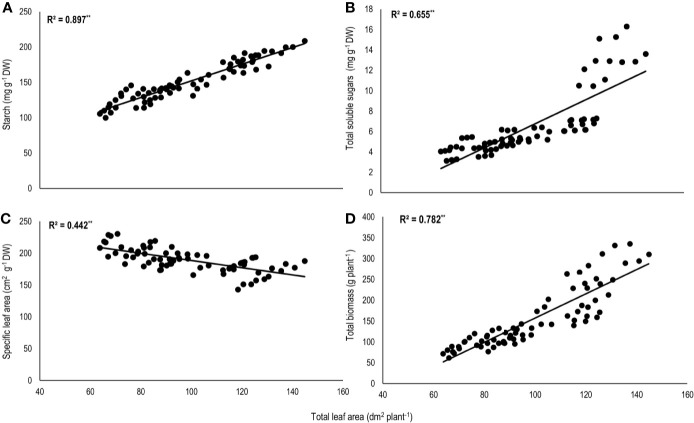
Relationship of total leaf area (TLA) with **(A)** starch, **(B)** total soluble sugars (TSS), **(C)** specific leaf area (SLA), and **(D)** total biomass (TB) of two sorghum hybrids (DK 53 and DK 28E) exposed to two mean temperatures (27 and 35°C) [27°C (optimum mean daytime and nighttime temperature; ODNT) and 35°C (high mean daytime and nighttime temperature; HDNT)] with three diurnal temperature amplitudes (2, 10, and 18°C). Coefficient of determination (R^2^) followed by ** significant at *P* < 0.01. Dry weight (DW).

Total biomass was positively correlated with starch (n=72; *P<*0.01; R^2^ = 0.77) and TSS (n=72; *P<*0.01; R^2^ = 0.73) while negatively with night respiration (n=72; *P<*0.01; R^2^ = 0.52) (**Figure 5**). Night respiration showed significant negative relationships with TSS (n=72; *P<*0.01; R^2^ = 0.49), RS (n=72; *P<*0.01; R^2^ = 0.23), NRS (n=72; *P<*0.01; R^2^ = 0.48) and starch (n=72; *P<*0.01; R^2^ = 0.73) (**Figures 6A–D**). TLA was positively correlated with starch (n=72; *P<*0.01; R^2^ = 0.90), TSS (n=72; *P<*0.01; R^2^ = 0.65) and TB (n=72; *P<*0.01; R^2^ = 0.78) and negatively with SLA (n=72; *P<*0.01; R^2^ = 0.44) (**Figures 7A–D**). Meanwhile, a weak relationship was observed between night respiration and *F*v/*F*m as well as with photosynthesis (data not shown). Night respiration showed negative correlation with TLA (n=72; *P<*0.01; R^2^ = 0.64) and positive correlation with SLA (n=72; *P<*0.01; R^2^ = 0.62) (**Supplementary Figure S1**). SLA was negatively correlated with starch (n=72; *P<*0.01; R^2^ = 0.41), TSS (n=72; *P<*0.01; R^2^ = 0.40) and TB (n=72; *P<*0.01; R^2^ = 0.33) (**Supplementary Figure S2**).

## Discussion

Our current study shows that narrowing of diurnal temperature amplitude (18 to 2°C) caused reduction in total leaf area and above ground total biomass along with an increase in night respiration and SLA and changes in carbohydrate composition [total soluble sugars, reducing sugars, non-reducing sugars and starch] in selected sorghum hybrids (**Figures 2****–****4**). However, photosynthesis was not affected with changes in diurnal temperature amplitude ranges (2, 10, and 18°C) tested in this study indicating altered carbon tradeoff (**Figure 3**). Overall response of physiological, biochemical and growth traits of sorghum hybrids were similar to those observed on maize (Sunoj et al., 2016). Growth traits of sorghum hybrids were severely reduced under HDNT (35°C) followed by ODNT (27°C) with narrow diurnal temperature amplitude of 2°C having lower daytime and higher nighttime temperatures as compared to diurnal temperature amplitudes of 10 and 18°C, except SLA. Simultaneously, opposite trend in growth traits was observed in wider diurnal temperature amplitude of 18°C, where temperature at daytime was high and nighttime was low (**Figure 2**). The impact of above contrasting daytime and nighttime temperatures of extremes of diurnal temperature amplitudes (2 and 18°C) on growth of sorghum hybrids specifies importance of diurnal temperature amplitude for sustaining optimum plant growth.

Light and dark phases in 24 h of a day are crucial for plant growth due active photosynthesis (CO_2_ assimilation) in daytime and respiration (CO_2_ release) in nighttime which are two components of foliar gas exchange correspondingly contributing and significant for carbon tradeoff and are inevitable for overall growth, development and yield (O’Leary and Plaxton, 2016; Sunoj et al., 2016). Despite similar rates of photosynthesis, there were significant differences in night respiration and total above ground biomass accumulation in both sorghum hybrids across all the deployed temperature conditions (ODNT and HDNT) in current study (**Figures 2** and **3**). This trend raises questions about the utilization of assimilated CO_2_ in the form of photosynthates and its end products (sucrose and starch) for growth and the influence of respiration. These differential responses in photosynthesis and above ground biomass accumulation point towards changes in night respiration and carbohydrate composition in response to temperature. However, there was also a strong relationship between total leaf area and biomass accumulation (**Figure 7**). Such an effect may arise from differential effects of temperature treatments on leaf area expansion (Tardieu et al., 1999), as consequent differences in leaf area will affect extent of light interception and hence, plant growth. This confounding effect of plant size needs to be considered when considering treatment effects on growth and biomass.

Correspondingly, Prasad et al., 2008 reported the same kind of response in photosynthetic performance of sorghum hybrid (DK 28E), in which, high day and night temperatures was set to 40/30°C (diurnal temperature amplitude of 10°C with mean temperature of 35°C and one of the day/night temperature condition used in the current study) and such response can be attributed to the ability of C_4_ crops to undergo thermal acclimation (Dwyer et al., 2007; Sage and Kubien, 2007; Wang et al., 2012). This was further supported by the stable photosynthesis in C_4_ crop maize under high temperature conditions (Sunoj et al., 2016; Rotundo et al., 2019). Additionally, previous studies on diverse C3 and C4 crops also demonstrated zero impact of high temperature on the subsequent day photosynthesis (potato: Lafta and Lorenzen, 1995; tomato: Huckstadt et al., 2013), while it has impact on night respiration, carbohydrate composition and growth (rice: Peraudeau et al., 2015; maize: Sunoj et al., 2016). Contrastingly, in C3 crop quinoa, photosynthesis and night respiration was increased under high temperature condition (Eustis et al., 2020).

Furthermore, decrease in photochemical efficiency of photosystem II (PSII) (*F*v/*F*m) is considered as indicator of photoinhibition or photodamage of PSII in plants under diverse abiotic stresses (Huang et al., 2016; Sunoj et al., 2016), varied across different temperature conditions (**Figure 3**). Besides, initial and reversible photoinhibition of PSII protects PSI from photoinhibition by limiting supply of electrons from PSII, which allow to keep the oxidative status of PSI under stress conditions. Hence, *F*v/*F*m values indicates health of leaves as well as total functionality of photosystems (PSII and PSI) (Joliot and Johnson, 2011; Takagi et al., 2017; Kadota et al., 2019). However, all the *F*v/*F*m values in both sorghum hybrids and temperature conditions in current study were equal or higher than 0.77 and this was only a slight change from the standard value (0.78) of *F*_v_/*F*_m_ representing uninterrupted functioning of PSII (Huang et al., 2016; Huang et al., 2018). The above minor reduction implies that photosystems (PSII and PSI) and linear electron flow (LEF) were slightly affected with narrow diurnal temperature amplitudes, even under HDNT. Nevertheless, the impact of variation in temperatures was not sufficient to tamper the light dependent reaction and thereby light independent (dark) reaction of photosynthesis in sorghum hybrids. It was one of the reasons resulted for and reflected from stable photosynthesis.

In plants, other than energy cost for ion uptake in roots and transport inside root and shoot, respiration [light phase (daytime) and dark phase (nighttime) respiration] has two main functions, which are maintenance and growth (Johnson, 1990; Atkin et al., 2000). Respiration is the controlled oxidation of energy-rich photosynthates or its end-products (sucrose and starch) synthesised from photosynthesis by the collective reactions of glycolysis, the tricarboxylic acid cycle and mitochondrial electron transport chain which produce CO_2_, adenosine 5-triphosphate (ATP), and low-molecular-weight molecules required for the biosynthesis of proteins and lipids and various metabolites essential for growth and plants to acclimation to stress (Atkin et al., 2000; O’Leary and Plaxton, 2016). Approximately, 30 to 70% of photosynthetically generated carbohydrates serve as major substrate as compared to the seldom used fatty acids that are utilized for respiration; and rate of respiration varies with plant species and status of growth conditions (optimum or sub or supra optimum) (Mohammed and Tarpley, 2011).

Respiration, especially at dark phase (night respiration), plays a significant role in recovery from injuries or damages caused due to the oxidative stress under optimum and diverse abiotic stress (moderate to severe injuries) conditions, in addition to contribution towards overall growth (Sunoj et al., 2016; Impa et al., 2018). However, depending on the extent of injuries or damage due to any type of stress, more resources are channelled for repair or maintenance respiration rather than contributing to growth, therefore, growth processes of the plants are negatively affected (Loka and Oosterhuis, 2010; Mohammed and Tarpley, 2011; Sunoj et al., 2016).

In current study, higher night respiration and reduction in assimilated total soluble sugars (reducing and non-reducing sugars), starch and biomass accumulation in both sorghum hybrids under narrowing diurnal temperature amplitude of ODNT and HDNT (**Figures 2****–****4**) imply higher level of carbohydrate utilization for the maintenance rather than growth. This was further evident from the negative correlations of night respiration with carbohydrate composition (**Figure 6**) and total biomass and positive correlation among total biomass and carbohydrate composition (**Figure 5**). Carbohydrate composition consist of glucose, fructose, and maltose which are reducing sugars, sucrose is major transported non-reducing sugar in plants and starch is a polysaccharide stored in carbon sinks of plants and synthesized from sucrose (Loka and Oosterhuis, 2010; Halford et al., 2011). Meanwhile, highest night respiration under HDNT with 2°C diurnal temperature amplitude (**Figure 3**) specifies that balance between two functions (maintenance and growth) of night respiration differs with the type and intensity of stress (Djanaguiraman et al., 2013; Sperling et al., 2015; Sunoj et al., 2016; Impa et al., 2018). High daytime and nighttime temperature reported to increase the night respiration and reduce ATP, carbohydrate composition and growth in diverse crops as well (cotton: Loka and Oosterhuis, 2010; soybean: Djanaguiraman et al., 2013; maize: Sunoj et al., 2016). In contrast, non-significant relatioship between night respiration and growth was also reported (tomato, soybean and lettuce: Frantz et al., 2004; rice: Peraudeau et al., 2015). At the same time, dark phase in plants are equally important as light phase not only due to contribution of night respiration but also because of its effect on synchronization of circadian clock to manage the redox state of photosystems, response of hormones and induction or control of different mechanisms which are collectively responsible for sustainable growth under optimum and tolerance under stress conditions (McClung, 2006; Sunoj et al., 2016; Srivastava et al., 2019).

Studies in sorghum have also shown a strong influence of temperature on leaf initiation, appearance, and expansion rates, all of which are important for development of plant leaf area and hence, light interception (LI) and biomass accumulation (Hammer and Muchow, 1994; Ravi Kumar et al., 2012). In the current experiment, overall, there was positive correlation of total leaf area with total biomass and carbohydrates (**Figure 7**). This indicates the contribution of plant size *via* leaf area to differences in biomass accumulation in addition to night respiration and utilization of carbohydrates (**Figures 3****–****6**), rather than from photosynthesis, which was stable across different temperature conditions (**Figure 3**). Progressive increases in total leaf area and biomass with increasing diurnal temperature amplitudes under both ODNT and HDNT further indicates the important role of leaf area in effecting biomass accumulation (**Figure 2**).

Some research has shown that leaf area expansion under different temperature conditions is independent of carbohydrate availability (Tardieu et al., 1999; Volkenburgh, 1999), so that SLA is a consequence of the temperature-driven potential leaf area expansion and available assimilates. SLA increases (i.e. more leaf area per unit dry weight) when environmental conditions have greater negative effect on assimilate availability than on leaf expansion rate (Tardieu et al., 1999). The negative correlation of SLA with total leaf area (**Figure 7**) indicates reduced availability of assimilates for plants that had been restricted in leaf area. The smaller plants found in narrow diurnal temperature amplitudes under both ODNT and HDNT (**Figure 2**) also had higher night respiration, which is consistent with their increased SLA and the overall positive association of SLA with night respiration (**Supplementary Figure S1**). Hence, it is likely that a combination of temperature effects on leaf expansion and night time respiration are generating the consequences observed on biomass accumulation. The importance of leaf area expansion on growth under diverse temperature conditions might also explain findings in studies in other crops where a nonsignificant relationship between night respiration and growth was reported (Frantz et al., 2004; Peraudeau et al., 2015).

Although the contribution of night respiration for growth may be confounded by effects on leaf expansion, understanding the crop and genotypic response of night respiration to the variation in day and night temperatures remains important. This is due to the fact that overall contribution of respiration is approximately 50% of the total annual CO_2_ input from the terrestrial ecosystem to atmosphere by taking part in plant growth and development (Gifford, 2003; O’Leary and Plaxton, 2016). Furthermore, our results indicate that effects of temperature on night respiration have a significant impact on biomass accumulation. This implies the importance of maintaining an optimum diurnal temperature amplitude that does not cause extreme maximum or minimum temperature is critical for attaining maximum growth and biomass.

## Conclusions

The results from the current study clearly demonstrated the importance of diurnal temperature amplitude on growth of sorghum. Relative to optimum mean daytime and nighttime temperature (27°C), high mean daytime and nighttime temperature (35°C) had negative impacts on physiology and growth of sorghum. Carbon tradeoff in sorghum was significantly altered under different diurnal temperatures amplitude with optimum or high mean temperatures. This was evident from stable CO_2_ assimilation (light phase photosynthesis), altered utilization (dark phase night respiration) and changes in total leaf area, specific leaf area and biomass accumulation and their relationships. Narrowing of diurnal temperature amplitude resulted increase in night respiration by utilizing the carbohydrate for maintenance rather than growth. These reveals the importance of understanding the dark phase mechanisms along with better understating the above ground biomass, leaf area components, and related light interception under different temperature conditions. Both hybrids monitored in current study to understand inter genotypic differences showed same pattern of response to diurnal temperature amplitudes. However, research with larger number of genotypes are needed to further confirm and understand genotypic response to different diurnal amplitude for the same mean temperature. Results from current study ascertain that impacts of diurnal temperature amplitude on leaf area, biomass accumulation and night respiration should be incorporated while quantifying the impact of temperature on growth and yield of crops. In addition, methods should consider appropriate temperature conditions and temperature amplitude while screening for temperature tolerant/sensitive genotypes. Furthermore, greater importance should be given to breed for genotypes/hybrids to maintain productivity and yield in areas facing or expecting high night temperatures and changing temperature amplitude. However, it is strongly recommended that separate studies should be conducted for different crops irrespective of their photosynthetic pathway (C_3_ or C_4_) under field conditions to achieve conclusions that are more realistic conditions without restrictions of root growth and natural light conditions. Finally, further research to understand optimum respiration under different temperature conditions will be important to identify crops or genotypes within crops that will be more suitable under changing climatic conditions.

## Data Availability Statement

The raw data supporting the conclusions of this article will be made available by the authors, without undue reservation.

## Author Contributions

VS, IC, and PP designed the research. VS and HM conducted the experiment, collected and analyzed the data and contributed to statistical analysis. VS and HM drafted the manuscript. PP and IC suggested critical comments and corrections while preparing manuscript. All authors contributed to the article and approved the submitted version.

## Funding

Financial support from Center for Sorghum Improvement at Kansas State University (KSU) and United States Agency for International Development (USAID) through Sustainable Agricultural and Natural Resource Management (SANREM) Collaborative Research Support Program for conducting the research. Preparation and publication of the manuscript was supported through Feed the Future Innovation Lab for Collaborative Research on Sustainable Intensification (Grant no. AID-OAA-L-14-00006).

## Conflict of Interest

The authors declare that the research was conducted in the absence of any commercial or financial relationships that could be construed as a potential conflict of interest.

## Supplementary Material

Supplementary Figure S1 Relationship of night respiration with **(A)** total leaf area (TLA) and **(B)** specific leaf area (SLA) of two sorghum hybrids (DK 53 and DK 28E) exposed to two mean temperatures (27 and 35°C) (27°C [optimum mean daytime and nighttime temperature; ODNT] and 35°C (high mean daytime and nighttime temperature; HDNT]) with three diurnal temperature amplitudes (2, 10, and 18°C). Coefficient of determination (R^2^) followed by ** corresponding to significance at *P*<0.01. Dry weight (DW).

Supplementary Figure S2 Relationship of specific leaf area (SLA) with **(A)** starch, **(B)** total soluble sugars (TSS) and **(C)** total biomass (TB) of two sorghum hybrids (DK 53 and DK 28E) exposed to two mean temperatures (27 and 35°C) (27°C [optimum mean daytime and nighttime temperature; ODNT] and 35°C (high mean daytime and nighttime temperature; HDNT]) with three diurnal temperature amplitudes (2, 10, and 18°C). Coefficient of determination (R^2^) followed by ** corresponding to significance at *P*<0.01. Dry weight (DW).

## Abbreviations

ODNT, optimum daytime and nighttime temperature; HDNT, high daytime and nighttime temperature; *F*v/*F*m, maximum photochemical efficiency of PSII; PAR, photosynthetic active radiation; TB, total aboveground biomass accumulation; TLA, total leaf area; SLA, Specific leaf area; TSS, total soluble sugars; RS, reducing sugars; NRS, non-reducing sugars.

## References

[B1] Atkin O. K. Millar A. H. Gardestrom P. Day D. A. (2000). “Photosynthesis, carbohydrate metabolism and respiration in leaves of higher plants,” in Photosynthesis: Physiology and Metabolism. Eds. Leegood R. C. Sharkey T. D. von Caemmerer S. A. (Netherlands: Academic Publishers), 153–175.

[B2] Bueno A. C. R. Prudente D. A. Machado E. C. Ribeiro R. V. (2012). Daily temperature amplitude affects the vegetative growth and carbon metabolism of orange trees in a rootstock-dependent manner. J. Plant Growth Regul. 31, 309–319. 10.1007/s00344-011-9240-x

[B3] Ciampitti I. A. Prasad P. V. V. (2020). Sorghum: State of art and future perspective (New Jersey, USA: John Wiley & Sons).

[B4] Djanaguiraman M. Prasad P. V. V. Schapaugh W. T. (2013). High day or nighttime temperature alters leaf assimilation reproductive success, and phosphatidic acid of pollen grain in soybean [*Glycine max* (L.) Merr.]. Crop Sci. 53, 1594–1604. 10.2135/cropsci2012.07.0441

[B5] Djanaguiraman M. Prasad P. V. V. Murugan M. Perumal R. Reddy U. K. (2014). Physiological differences among sorghum (*Sorghum bicolor* L., Moench) genotypes under high temperature stress. Environ. Exp. Bot. 100, 43–54. 10.1016/j.envexpbot.2013.11.013

[B6] Djanaguiraman M. Perumal R. Jagadish S. V. K. Ciampitti I. A. Welti R. Prasad P. V. V. (2018a). Sensitivity of sorghum pollen and pistil to high-temperature stress. Plant Cell Environ. 41, 1065–1082. 10.1111/pce.13089 29044571 PMC5904002

[B7] Djanaguiraman M. Perumal R. Ciampitti I. A. Gupta S. K. Prasad P. V. V. (2018b). Quantifying pearl millet response to high temperature stress: thresholds, sensitive stages, genetic variability and relative sensitivity of pollen and pistil. Plant Cell Environ. 41, 993–1007. 10.1111/pce.12931 28173611

[B8] Dubois M. Gilles K. A. Hamilton J. K. Rebers P. A. Smith F. (1956). Colorimetric method for determination of sugars and related substances. Anal. Chem. 28, 350–356. 10.1021/ac60111a017

[B9] Dwyer S. A. Ghannoum O. A. Caemmerer S. V. (2007). High temperature acclimation of C_4_ photosynthesis is linked to changes in photosynthetic biochemistry. Plant Cell Environ. 30, 53–66. 10.1111/j.1365-3040.2006.01605.x 17177876

[B10] Eustis A. Murphy K. M. Barrios-Masias F. H. (2020). Leaf gas exchange performance of ten quinoa genotypes under a simulated heat wave. Plants 9:81. 10.3390/plants9010081 PMC7020487 31936466

[B11] Frantz M. J. Cometti N. N. Bugbee B. (2004). Night temperature has a minimal effect on respiration and growth in rapidly growing plants. Ann. Bot. 94, 155–166. 10.1093/aob/mch122 15159217 PMC4242378

[B12] Gifford R. M. (2003). Plant respiration in productivity models: conceptualization, representation and issues for global terrestrial carbon-cycle research. Funct. Plant Biol. 30, 171–186. 10.1071/FP02083 32689003

[B13] Halford N. G. Curtis T. Y. Muttucumaru N. Postles J. Mottram D. S. (2011). Sugars in crop plants. Ann. Appl. Biol. 158, 1–25. 10.1111/j.1744-7348.2010.00443.x

[B14] Hammer G. L. Muchow R. C. (1994). Assessing climatic risk to sorghum production in water-limited subtropical environments. I. Development and testing of a simulation model. Field Crops Res. 36, 221–234. 10.1016/0378-4290(94)90114-7

[B15] Hammer G. L. Carberry P. S. Muchow R. C. (1993). ). Modeling genotypic and environmental control of leaf area dynamics in grain sorghum. I. Whole plant level. Field Crops Res. 33, 293–310. 10.1016/0378-4290(93)90087-4

[B16] Hedge J. E. Hofreiter B. T. (1962). “Estimation of carbohydrate,” in Methods in Carbohydrate Chemistry. Eds. Whistler R. L. Be Miller J. N. (New York, USA: Academic Press), 17–22.

[B17] Huang W. Yang Y. Hu H. Cao K. F. Zhang S. (2016). Sustained diurnal stimulation of cyclic electron flow in two tropical tree species *Erythrophleum guineense* and *Khaya ivorensis*. Front. Plant Sci. 7, 1068. 10.3389/fpls.2016.01068. Article 1068. 27486473 PMC4950474

[B18] Huang W. Quan X. Zhang S. B. Liu T. (2018). In vivo regulation of proton motive force during photosynthetic induction. Environ. Exp. Bot. 148, 109–116. 10.1016/j.envexpbot.2018.01.001

[B19] Huckstadt A. B. Suthaparan A. Mortensen L. M. Gislerod H. R. (2013). The effect of low night and high day temperatures on photosynthesis in tomato. Am. J. Plant Sci. 4, 2323–2331. 10.4236/ajps.2013.412288

[B20] Impa M. S. Sunoj V. S. J. Krassovskaya I. Raju B. R. Obata T. Jagadish S. V. K. (2018). Carbon balance and source-sink metabolic changes in winter wheat exposed to high night-time temperature. Plant Cell Environ. 42, 1233–1246. 10.1111/pce.13488 30471235

[B21] Inthichack P. Nishimura Y. Fukumoto Y. (2013). Diurnal temperature alternations on plant growth and mineral absorption in eggplant, sweet pepper, and tomato. Hortic. Environ. Biotechnol. 54, 37–43. 10.1007/s13580-013-0106-y

[B22] Inthichack P. Nishimura Y. Fukumoto Y. (2014). Effect of diurnal amplitude on plant growth and mineral composition in cucumber, melon and water melon. Pak. J. Biol. Sci. 17, 1030–1036. 10.3923/pjbs.2014.1030.1036 26031022

[B23] IPCC (2018). Summary for Policymakers” in Global warming of 1.5°C. An IPCC Special Report on the impacts of global warming of 1.5°C above pre-industrial levels and related global greenhouse gas emission pathways, in the context of strengthening the global response to the threat of climate change, sustainable development, and efforts to eradicate poverty. Eds. Masson-Delmotte V. Zhai P. Pörtner H. O. Roberts D. Skea J. Shukla P. R. Pirani A. Moufouma-Okia W. Péan C. Pidcock R. Connors S. Matthews J. B. R. Chen Y. Zhou X. Gomis M. II Lonnoy E. Maycock T. Tignor M. Waterfield T. (Geneva, Switzerland: World Meteorological Organization).

[B24] Johnson I. R. (1990). Plant respiration in relation to growth, maintenance, ion uptake and nitrogen assimilation. Plant Cell Environ. 13, 319–328. 10.1111/j.1365-3040.1990.tb02135.x

[B25] Joliot P. Johnson G. (2011). Regulation of cyclic and linear electron flow in higher plants. Proc. Natl. Acad. Sci. U.S.A. 108, 13317–13322. 10.1073/pnas.1110189108 21784980 PMC3156182

[B26] Kadota K. Furutani R. Makino A. Suzuki Y. Wada S. Miyake C. (2019). Oxidation of P700 induces alternative electron flow in photosystem I in wheat leaves. Plants 8, 152. 10.3390/plants8060152 PMC6631986 31195693

[B27] Lafta A. M. Lorenzen J. H. (1995). Effect of high temperature on plant growth and carbohydrate metabolism in potato. Plant Physiol. 109, 637–643. 10.1104/pp.109.2.637 12228617 PMC157630

[B28] Lin G. Yang Y. Chen X. Yu X. Wu Y. Xiong F. (2020). Effects of high temperature during two growth stages on caryopsis development and physicochemical properties of starch in rice. Int. J. Biol. Macromol. 14, 301–310. 10.1016/j.ijbiomac.2019.12.190 31874272

[B29] Lizaso J. I. Ruiz-Ramosa M. Rodrigueza L. Gabaldon-Lealb C. Oliveirac J. A. Loriteb I. J. (2018). Impact of high temperatures in maize: phenology and yield components. Field Crops Res. 216, 129–140. 10.1016/j.fcr.2017.11.013

[B30] Lobell D. B. Sibley A. Ortiz Monasterio J. I. (2012). Extreme heat effects on wheat senescence in India. Nat. Clim. Change 2, 186–189. 10.1038/nclimate1356

[B31] Loka D. A. Oosterhuis D. M. (2010). Effect of high night temperatures on cotton respiration: ATP levels and carbohydrate content. Environ. Exp. Bot. 68, 258–263. 10.1016/j.envexpbot.2010.01.006

[B32] Luo Q. (2011). Temperature thresholds and crop production: a review. Climatic Change 109, 583–598. 10.1007/s10584-011-0028-6

[B33] Maikasuwa M. A. Ala A. L. (2013). Trend analysis of area and productivity of sorghum in Sokoto state, Nigeria 1993–2012. Eur. Sci. J. 9, 69–75. 10.19044/esj.2013.v9n16p%p

[B34] Maiti R. K. (1996). Sorghum Science. Lebanon, NH, USA: Science Publishers.

[B35] Malhotra S. S. Sarkar S. K. (1979). Effects of sulphur dioxide on sugar and free amino acid content of pine seedlings. Physiol. Plant 47, 223–228.

[B36] Maswada F. H. Sunoj V. S. J. Prasad P. V. V. (2020). A comparative study on the effect of seed pre-sowing treatments with microwave radiation and salicylic acid in alleviating the drought-induced damage in wheat. J. Plant Growth Regul. 10.1007/s00344-020-10079-3

[B37] Matsuda R. Ozawa N. Fujiwara K. (2014). Leaf photosynthesis plant growth, and carbohydrate accumulation of tomato under different photoperiods and diurnal temperature differences. Sci. Hortic. 170, 150–158. 10.1016/j.scienta.2014.03.014

[B38] McClung C. R. (2006). Historical perspective essay: Plant circadian rhythm. Plant Cell 8, 792–803. 10.1105/tpc.106.040980 PMC1425852 16595397

[B39] Mohammed A. K. Tarpley L. (2009). Impact of high nighttime temperature on respiration membrane stability, antioxidant capacity, and yield of rice plants. Crop Sci. 49, 313–322. 10.2135/cropsci2008.03.0161

[B40] Mohammed A. K. Tarpley L. (2011). ““Effects of high night temperature on crop physiology and productivity,” in Plant growth regulators provide a management option, global warming impacts – case studies on the economy, human health, and on urban and natural environments. Ed. Casalegno S., (IntechOpen). 10.5772/24537

[B41] Myster J. Moe R. (1995). Effect of diurnal temperature alternations on plant morphology in some greenhouse crops - A mini review. Sci. Hortic. 62, 205–215. 10.1016/0304-4238(95)00783-P

[B42] Narayanan S. Prasad P. V. V. Fritz A. K. Boyle D. L. Gill B. S. (2015). Impact of high night-time and high daytime temperature stress on winter wheat. J. Agron. Crop Sci. 201, 206–218. 10.1111/jac.12101

[B43] Narayanan S. Prasad P. V. V. Welti R. (2018). Alterations in wheat pollen lipidome during high day and night temperature stress. Plant Cell Environ. 41, 1749–1761. 29377219 10.1111/pce.13156 PMC6713575

[B44] Opole R. A. Prasad P. V. V. Djanaguiraman M. Vimala K. Kirkham M. B. Upadhyaya H. D. (2018). Thresholds, sensitive stages and genetic variability of finger millet to high temperature stress. J. Agron. Crop Sci. 204, 477–492. 10.1111/jac.12279

[B45] O’Leary B. M. Plaxton W. C. (2016). “Plant Respiration” (New Jersey, USA: John Willy & Sons).

[B46] Peng S. Huang J. Sheehy J. E. Laza R. C. Visperas R. M. Zhong X. (2004). Rice yields decline with higher night temperature from global warming. Proc. Natl. Acad. Sci. 101, 9971–9975. 10.1073/pnas.0403720101 15226500 PMC454199

[B47] Peraudeau S. Lafarge T. Roques S. Quinones C. O. Clement-Vidal A. Ouwerkerk P. B. F. (2015). Effect of carbohydrates and night temperature on night respiration in rice. J. Exp. Bot. 66, 3931–3944. 10.1093/jxb/erv193 25954047

[B48] Prasad P. V. V. Djanaguiraman M. (2011). High night temperature decreases leaf photosynthesis and pollen function in grain sorghum. Funct. Plant Biol. 38, 993–1003. 10.1071/FP11035 32480957

[B49] Prasad P. V. V. Boote K. J. Allen L. H. Jr Sheehy J. E. Thomas J. M. G. (2006a). Species, ecotypes and cultivar differences in spikelet fertility and harvest index of rice in response to high temperature stress. Field Crops Res. 95, 398–411. 10.1016/j.fcr.2005.04.008

[B50] Prasad P. V. V. Boote K. J. Allen L. H. Jr (2006b). Adverse high temperature effects on pollen viability, seed-set, seed yield and harvest index of grain-sorghum [*Sorghum bicolor* (L.) Moench] are more severe at elevated carbon dioxide due to high tissue temperature. Agric. Forest. Meteorol. 139, 237–251. 10.1016/j.agrformet.2006.07.003

[B51] Prasad P. V. V. Pisipati S. Mutava R. N. Tuinstra M. R. (2008a). Sensitivity of grain sorghum to high temperature stress during reproductive development. Crop Sci. 48, 1911–1917. 10.2135/cropsci2008.01.0036

[B52] Prasad P. V. V. Pisipati S. R. Ristic Z. Bukovnik U. Fritz A. K. (2008b). Impact of nighttime temperature on physiology and growth of spring wheat. Crop Sci. 48, 2372–2380. 10.2135/cropsci2007.12.0717

[B53] Prasad P. V. V. Djanaguiraman M. Perumal R. Ciampitti I. A. (2015). Impact of high temperature stress on floret fertility and individual grain weight of grain sorghum: Sensitive stages and thresholds for temperature and duration. Front. Plant Sci. 6, 820. 10.3389/fpls.2015.00820 26500664 PMC4594118

[B54] Ravi Kumar S. Hammer G. L. Broad I. Harland P. McLean G. (2009). Modelling environmental effects on phenology and canopy development of diverse sorghum genotypes. Field Crops Res. 111, 157–165. 10.1016/j.fcr.2008.11.010

[B55] Roozeboom K. L. Prasad P. V. V. (2016). “Sorghum Growth and Development,” in Sorghum: State of art and future perspective. Eds. Ciampitti I. A. Prasad P. V. V. (New Jersey, USA: John Willy & Sons), 1–18.

[B56] Rotundo J. L. Tang T. Messina C. D. (2019). Response of maize photosynthesis to high temperature: Implications for modeling the impact of global warming. Plant Physiol. Biochem. 141, 202–205. 10.1016/j.plaphy.2019.05.035 31176879

[B57] Sage R. F. Kubien D. S. (2007). The temperature response of C_3_ and C_4_ photosynthesis. Plant Cell Environ. 30, 1086–1106. 10.1111/j.1365-3040.2007.01682.x 17661749

[B58] Singh V. Nguyen C. T. van Oosterom E. J. Chapman S. C. Jordan D. R. Hammer G. L. (2015). Sorghum genotypes differ in high temperature responses for seed set. Field Crops Res. 171, 32–40. 10.1016/j.fcr.2014.11.003

[B59] Somogyi M. (1952). Estimation of sugars by colorimetric method. J. Biol. Chem. 200, 245.

[B60] Sperling O. Earles J. M. Secchi F. Godfrey J. Zwieniecki M. A. (2015). Frost induces respiration and accelerates carbon depletion in trees. PloS One 10 (12), e0144124. 10.1371/journal.pone.0144124 26629819 PMC4668004

[B61] Srivastava D. Shamim M. Kumar M. Mishra A. Maurya R. Sharma D. (2019). Role of circadian rhythm in plant system: An update from development to stress response. Environ. Exp. Bot. 162, 256–271. 10.1016/j.envexpbot.2019.02.025

[B62] Sunoj V. S. J. Shroyer K. J. Jagadish S. V. K. Prasad P. V. V. (2016). Diurnal temperature amplitude alters physiological and growth response of maize (*Zea mays* L.) during the vegetative stage. Environ. Exp. Bot. 130, 113–121. 10.1016/j.envexpbot.2016.04.007

[B63] Sunoj V. S. J. Impa M. S. Chiluwal A. Perumal R. Prasad P. V. V. Jagadish S. V. K. (2017). Resilience of pollen and post flowering response in diverse sorghum genotypes exposed to heat stress under field conditions. Crop Sci. 57, 1–12. 10.2135/cropsci2016.08.0706

[B64] Takagi D. Ishizaki K. Hanawa H. Mabuchi T. Shimakawa G. Yamamotod H. (2017). Diversity of strategies for escaping reactive oxygen species production within photosystem I among land plants: P700 oxidation system is prerequisite for alleviating photoinhibition in photosystem I. Physiol. Plant 161, 56–74. 10.1111/ppl.12562 28295410

[B65] Tardieu F. Granier C. Muller B. (1999). Research review: modelling leaf expansion in a fluctuating environment: are changes in specific leaf area a consequence of changes in expansion rate? New Phytol. 143, 33–43. 10.1046/j.1469-8137.1999.00433.x

[B66] Tari I. Laskay G. Takacs Z. Poor P. (2013). Response of sorghum to abiotic stresses: A review. J. Agron. Crop Sci. 199, 264–274. 10.1111/jac.12017

[B67] Volkenburgh E. V. (1999). Leaf expansion-An integrating plant behavior. Plant Cell Environ. 22, 1463–1473. 10.1046/j.1365-3040.1999.00514.x

[B68] Vose R. S. Easterling D. R. Gleason B. (2005). Maximum and minimum temperature trends for the globe: an update through 2004. Geophys. Res. 32, 23822. 10.1029/2005GL024379

[B69] Wang C. Guo L. Li Y. Wang Z. (2012). Systematic comparison of C_3_ and C_4_ plants based on metabolic network analysis. BMC Syst. Biol. 6 (2), S9. 10.1186/1752-0509-6-S2-S9 PMC3521184 23281598

[B70] Wang K. Li Y. Wang Y. Yang X. (2017). On the asymmetry of urban daily air temperature cycle. J. Geophys. Res. Atmos. 122, 5625–5635. 10.1002/2017JD026589

[B71] Wang Y. Zhang Y. Shi Q. Chen H. Xiang J. Hu G. (2020). Decrement of sugar consumption in rice young panicle under high temperature aggravates spikelet number reduction. Rice Sci. 27, 44–55. 10.1016/j.rsci.2019.12.005

[B72] Welch J. R. Vincent J. R. Auffhammer M. Moya P. F. Dobermann A. Dawe D. (2010). Rice yields in tropical/subtropical Asia exhibit large but opposing sensitivities to minimum and maximum temperatures. Proc. Natl. Acad. Sci. 33, 14562–14567. 10.1073/pnas.1001222107 PMC2930450 20696908

[B73] Xiong J. Patil G. G. Moe R. Torre S. (2011). Effects of diurnal temperature alternations and light quality on growth, morphogenesis and carbohydrate content of *Cucumis sativus* L. Sci. Hortic. 128, 54–60. 10.1016/j.scienta.2010.12.013

