# Supplementary figures and images for: Narrowing Diurnal Temperature Amplitude Alters Carbon Tradeoff and Reduces Growth in C4 Crop Sorghum
Source: Front Plant Sci. 2020 Aug 19;11:1262. doi: 10.3389/fpls.2020.01262 (PMC7466774; doi:10.3389/fpls.2020.01262)

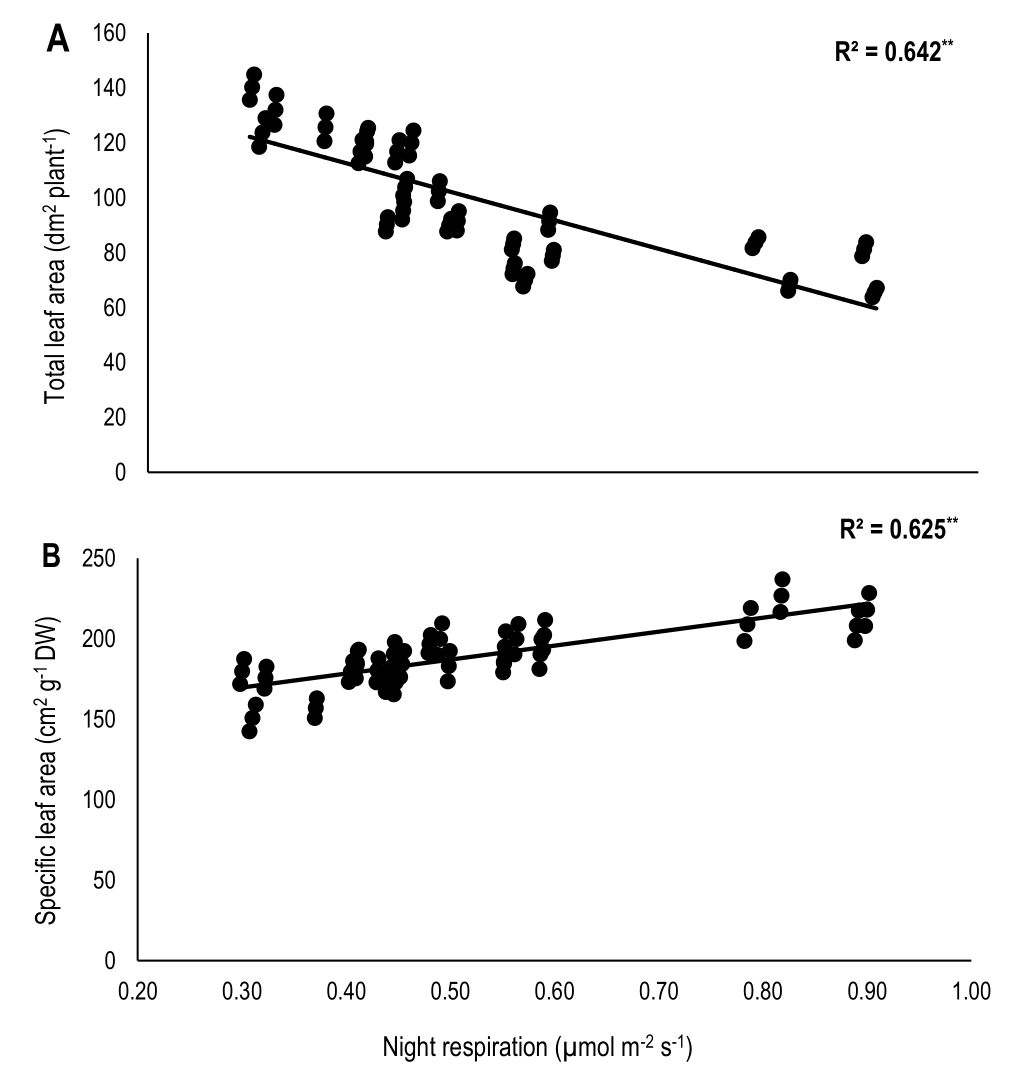

Supplement: Supplementary Figure S1 — Relationship of night respiration with (A) total leaf area (TLA) and (B) specific leaf area (SLA) of two sorghum hybrids (DK 53 and DK 28E) exposed to two mean temperatures (27 and 35°C) (27°C [optimum mean daytime and nighttime temperature; ODNT] and 35°C (high mean daytime and nighttime temperature; HDNT]) with three diurnal temperature amplitudes (2, 10, and 18°C). Coefficient of determination (R2) followed by ** corresponding to significance at P<0.01. Dry weight (DW). [file Image_1.tif]

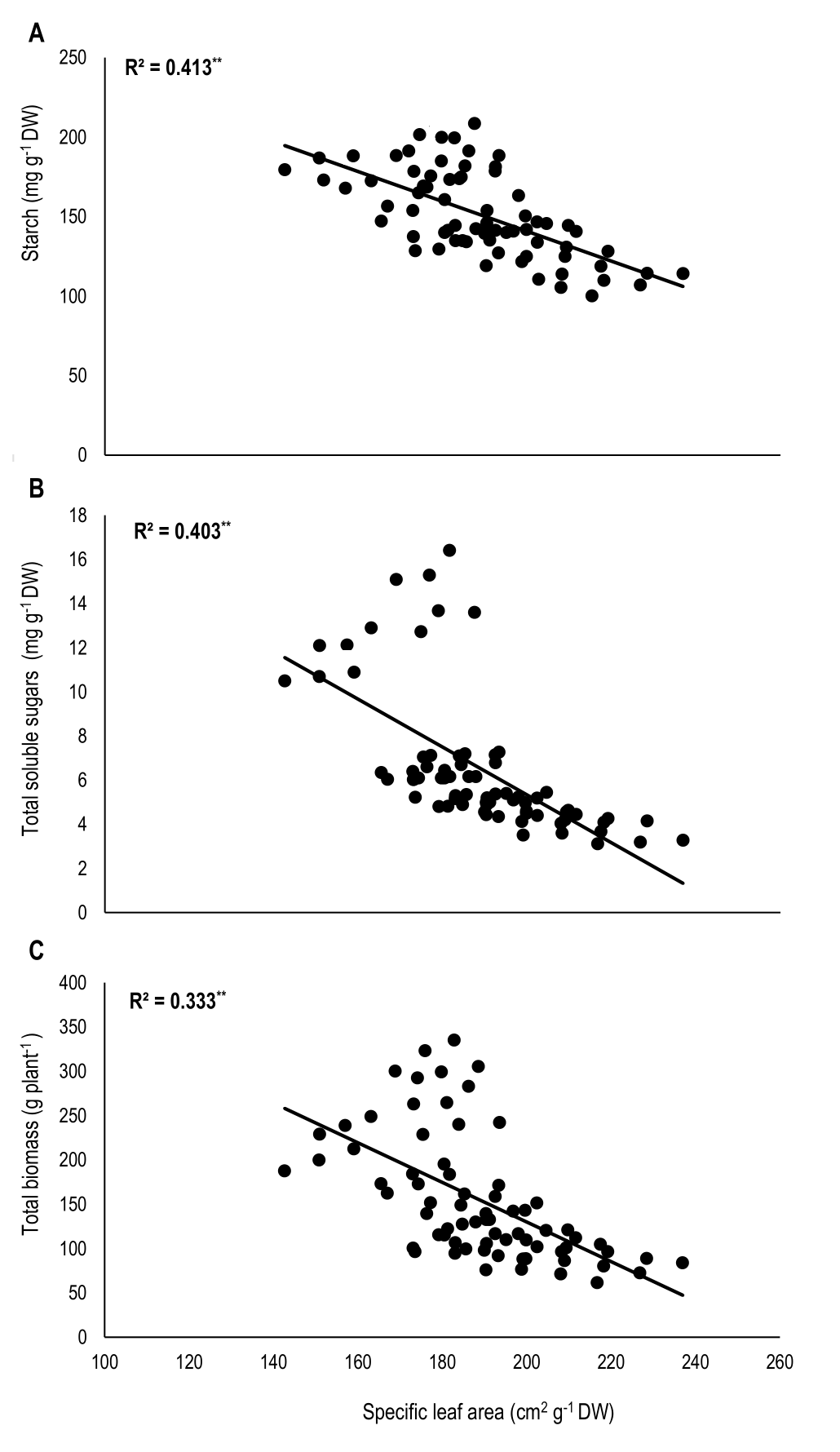

Supplement: Supplementary Figure S2 — Relationship of specific leaf area (SLA) with (A) starch, (B) total soluble sugars (TSS) and (C) total biomass (TB) of two sorghum hybrids (DK 53 and DK 28E) exposed to two mean temperatures (27 and 35°C) (27°C [optimum mean daytime and nighttime temperature; ODNT] and 35°C (high mean daytime and nighttime temperature; HDNT]) with three diurnal temperature amplitudes (2, 10, and 18°C). Coefficient of determination (R2) followed by ** corresponding to significance at P<0.01. Dry weight (DW). [file Image_2.tif]
